# Mistreatment of women during childbirth in Abuja, Nigeria: a qualitative study on perceptions and experiences of women and healthcare providers

**DOI:** 10.1186/s12978-016-0265-2

**Published:** 2017-01-17

**Authors:** Meghan A. Bohren, Joshua P. Vogel, Özge Tunçalp, Bukola Fawole, Musibau A. Titiloye, Akinpelu Olanrewaju Olutayo, Modupe Ogunlade, Agnes A. Oyeniran, Olubunmi R. Osunsan, Loveth Metiboba, Hadiza A. Idris, Francis E. Alu, Olufemi T. Oladapo, A. Metin Gülmezoglu, Michelle J. Hindin

**Affiliations:** 1 Department of Population, Family and Reproductive Health, Johns Hopkins Bloomberg School of Public Health, 615 N. Wolfe St, Baltimore, MD USA; 2 UNDP/UNFPA/UNICEF/WHO/World Bank Special Programme of Research, Development and Research Training in Human Reproduction (HRP), Department of Reproductive Health and Research, World Health Organization, Geneva, Switzerland; 3 Department of Obstetrics & Gynaecology, National Institute of Maternal & Child Health, College of Medicine, University of Ibadan, Ibadan, Nigeria; 4 Department of Health Promotion and Education, Faculty of Public Health, College of Medicine, University of Ibadan, Ibadan, Nigeria; 5 Department of Sociology, Faculty of the Social Sciences, University of Ibadan, Ibadan, Nigeria; 6 Nyanya General Hospital, Abuja, Federal Capital Territory Nigeria; 7 Maitama District Hospital, Abuja, Federal Capital Territory Nigeria

**Keywords:** Maternal health, Obstetric delivery, Childbirth, Mistreatment, Disrespect, Abuse, Quality of care, Qualitative research, Nigeria

## Abstract

**Background:**

Global efforts have increased facility-based childbirth, but substantial barriers remain in some settings. In Nigeria, women report that poor provider attitudes influence their use of maternal health services. Evidence also suggests that women in Nigeria may experience mistreatment during childbirth; however, there is limited understanding of how and why mistreatment this occurs. This study uses qualitative methods to explore women and providers’ experiences and perceptions of mistreatment during childbirth in two health facilities and catchment areas in Abuja, Nigeria.

**Methods:**

In-depth interviews (IDIs) and focus group discussions (FGDs) were used with a purposive sample of women of reproductive age, midwives, doctors and facility administrators. Instruments were semi-structured discussion guides. Participants were asked about their experiences and perceptions of, and perceived factors influencing mistreatment during childbirth. Thematic analysis was used to synthesize findings into meaningful sub-themes, narrative text and illustrative quotations, which were interpreted within the context of this study and an existing typology of mistreatment during childbirth.

**Results:**

Women and providers reported experiencing or witnessing physical abuse including slapping, physical restraint to a delivery bed, and detainment in the hospital and verbal abuse, such as shouting and threatening women with physical abuse. Women sometimes overcame tremendous barriers to reach a hospital, only to give birth on the floor, unattended by a provider. Participants identified three main factors contributing to mistreatment: poor provider attitudes, women’s behavior, and health systems constraints.

**Conclusions:**

Moving forward, findings from this study must be communicated to key stakeholders at the study facilities. Measurement tools to assess how often mistreatment occurs and in what manner must be developed for monitoring and evaluation. Any intervention to prevent mistreatment will need to be multifaceted, and implementers should consider lessons learned from related interventions, such as increasing audit and feedback including from women, promoting labor companionship and encouraging stress-coping training for providers.

## Plain English summary

In developing countries, 300,000 women per year die from complications during pregnancy and childbirth, and approximately fifteen percent of these deaths occur in Nigeria, West Africa. Most of these deaths could be avoided with access to good quality reproductive health services. In Nigeria, women may expect to receive poor quality of care at health facilities during pregnancy and childbirth, which may mean that they will not use these potentially life-saving services. Previous research suggests that women in Nigeria may be mistreated during childbirth in health facilities, including being slapped, pinched, yelled at, and neglected by health providers. In this study, we used qualitative research methods (in-depth interviews and focus group discussions) to explore the perceptions and experiences of mistreatment during childbirth, from the perspectives of women and health providers. The use of qualitative methods encouraged the study participants to share personal experiences in their own words, in order to better understand mistreatment during childbirth in Nigeria. We found that both women and health providers reported experiencing or witnessing physical abuse (such as slapping, physical restraint to a delivery bed, and detainment in the hospital when unable to pay bills) and verbal abuse (such as shouting and threatening women with physical abuse). Women sometimes overcame barriers to reach a hospital, only to give birth on the floor, unattended by a provider. These results will be used to start a discussion with health providers and communities on how to develop interventions to stop mistreatment during childbirth from happening in Nigeria.

## Background

An estimated 303,000 maternal deaths occurred in 2015, with 66.3% occurring in Sub-Saharan Africa [1]. While substantial progress has been made to reduce maternal mortality, one in 38 women residing in sub-Saharan Africa are still at risk of maternal death [2]. The majority of maternal deaths are preventable and manageable with good quality reproductive health services and skilled birth attendance. However, only 68% of deliveries in developing countries were attended by skilled birth attendants in 2012 [3], and only 43% were in facilities [4].

According to Demographic and Health Survey (DHS) estimates, the total fertility rate (TFR) in Nigeria in 2013 was 5.5, a slight drop from 6.4 in 1960 [5]. Nigeria accounts for 15% of the global burden of maternal mortality, with approximately 45,000 maternal deaths per year, and women in Nigeria have a 1 in 22 lifetime risk of maternal death [1]. Vast geographical health disparities exist, with poor health indicators in the northern regions compared to the southern regions, including an almost ten-fold difference in maternal mortality [5]. Poor use of maternal health services in Nigeria is a key factor contributing to high levels of maternal morbidity and mortality, as only 51.1% of women completed four or more antenatal care visits and only 36% of births took place in a health facility in 2013 [5]. Poor perceived quality of care at facilities is a critical barrier [6–10], and poor health worker attitudes contribute to a woman’s choice of using a facility or traditional provider [9, 11, 12]. A study from northwestern Nigeria concluded that 23.7% of women who did not give birth in a health facility cited negative provider attitudes as the primary reason for not using delivery services, and 52.0% of women suggested that improvements in provider attitudes are necessary to increase demand for facility-based childbirth [6]. Another study in southern Nigeria showed that women viewed government facilities as providing poor quality maternity services and had poor availability of trained staff during childbirth [11].

While there are global efforts to increase facility-based childbirth, there are significant barriers in some settings, preventing women from attending facilities, including distance [13, 14], cost [15, 16], and perceived quality of care [15, 17, 18]. More recently, improving quality of care, including women’s experiences of care, has been highlighted as a key component of strategies to further reduce preventable maternal mortality and morbidity [19]. However, recent evidence suggests that many women experience mistreatment during childbirth in health facilities across the world [20–29]. The terminology used in different parts of the world to describe the poor treatment of women during childbirth is variable and includes “obstetric violence” [30–32], “disrespect and abuse” [33–38] and “dehumanized care” [39, 40]. In this study, we use the terminology “mistreatment of women during childbirth” as a more inclusive term to “better capture the full range of experiences women and healthcare providers” have described. This includes intentional abuse, unintentional or passive abuse, and mistreatment resulting from both individual behaviors that constitute acts of mistreatment and health systems conditions that either women may experience as mistreatment or are drivers of mistreatment [29].

In 2010, Bowser and Hill published a landscape analysis that explored the evidence for “disrespect and abuse” during facility-based childbirth, and proposed a model to categorize types of abuse [41]. Using the categories proposed by Bowser and Hill, four recent studies in sub-Saharan Africa have measured disrespect and abuse through direct labor observations, facility exit interviews and community-based follow-up surveys [36–38, 42]. However, differential operational definitions, lack of consensus on what constitutes poor treatment and varying study designs have resulted in wide differences in prevalence, and it is unclear if differences in prevalence relate to differences in methodology or true variation [43]. These studies have highlighted that many women are mistreated during childbirth, but limitations exist to define and measure mistreatment during childbirth in a systematic and standardized way.

Developing an evidence-based typology of what constitutes mistreatment during childbirth was a critical next step. Therefore, a mixed-methods systematic review synthesized 65 studies conducted across 34 countries, and classified mistreatment into seven categories: physical, sexual and verbal abuse, stigma and discrimination, failure to meet professional standards of care, poor rapport between women and providers, and health systems conditions and constraints [29]. Khosla and colleagues have also described mistreatment during childbirth as a human rights violation [44]. Currently, a two-phased, mixed-methods study on mistreatment during childbirth is underway in Nigeria, Ghana, Guinea and Myanmar. In short, the first phase of this study is a formative phase consisting of a multi-country primary qualitative study [43]. Findings from the formative phase will improve understanding of both women’s and providers’ perspectives of mistreatment during childbirth, contributing factors,, identify potential entry points to reduce mistreatment, and inform the development of measurement tools to be used in the second phase. In this study, we used qualitative methods to explore women and healthcare providers’ experiences and perceptions of mistreatment during childbirth in facilities in the Abuja metropolitan area of Nigeria. The typology of mistreatment during childbirth [29] provided a framework in which to organize and present our findings of contextually-specific evidence of mistreatment during childbirth in Nigeria.

## Methods

### Study sites

This study was conducted in two communities in the Federal Capital Territory (one peri-urban/rural and one urban), in the north central region where approximately 45.7% of women gave birth in a facility in 2013 [5]. In the north central region, the median age at first marriage is 19.1 years (among women aged 20-49 years) and the total fertility rate is 5.3 [5]. Study facilities were chosen in collaboration with the local principal investigator using pre-specified inclusion criteria, including number of deliveries per month, number of staff currently employed, and an existing relationship between the research institution and the selected facilities. Characteristics of the study sites are shown in Table 1.

**Table 1:** Facility characteristics

	Peri-urban facility	Urban facility
Staffing		
Obstetrician/gynecologist	3	4
Medical officer	8	10
Midwife	15	12
Capacity		
# beds on delivery ward	6	4
Health outcomes (2013)		
Total births (n)	3231	2417
Live births (n)	2961	2182
Stillbirths (n)	270	235
Maternal deaths (n)	94	73
Cost of childbirth services		
Vaginal delivery	$0 USD	$0 USD
Caesarean section	$215 USD (42,000 NGN)	$215 USD (42,000 NGN)

Note: facility characteristics as reported by the head of each facility in personal communication, August 2014

### Study participants, recruitment and sampling

Three groups of participants were identified for this study: (1) women; (2) healthcare providers; and (3) facility administrators. FGDs were conducted with women of reproductive age (15–49 years) who gave birth in any facility in the past 5 years and resided in the selected facility catchment area. IDIs were conducted with women of reproductive age (15–49 years) who gave birth in a facility in the past 12 months and resided in the selected facility catchment area. We chose to conduct FGDs with women with a facility-based birth in the previous 5 years to allow for potential participants who experienced a subsequent birth outside the facility to be included in the study, as we hypothesized that poor treatment could influence future birth location. Women were ineligible to participate if they did not reside in the facility catchment area or did not give birth at any health facility in the past 12 months (IDIs) or 5 years (FGDs). Eligible women were allowed to participate in either an IDI or FGD, but not both. IDIs were conducted with healthcare providers (nurses/midwives and doctors/specialists) and facility administrators (e.g.: medical director, head of obstetrics, matron-in-charge). Healthcare providers were ineligible to participate if they did not work on the maternity ward of the study facilities. Both IDIs and FGDs were conducted with women in order to gain a detailed understanding of experiences of mistreatment during childbirth (IDIs) and to better understand social norms related to mistreatment (FGDs). Only IDIs were conducted with providers and administrators, due to concerns that FGDs may breach the confidentiality of study participants through the disclosure of poor practices or “naming and blaming”.

An obstetrician and midwife from each selected facility who attended the study training workshop acted as an entry point to connect research assistants to healthcare providers. Community health workers helped to identify women who met the inclusion criteria, and research assistants initiated face-to-face contact with women health care providers meeting the inclusion criteria. Each eligible individual was invited to participate and provide consent.

Quota sampling was used to achieve a stratified purposive sample without random selection using specified parameters to stratify the sample, including setting, religion, age and cadre. Women were sampled from the urban and rural/peri-urban communities in the selected facility catchment area, and were recruited based on their age/parity/religion in order to explore the experiences of both younger/primiparous and older/multiparous women. Although further stratification did not take place across ethnicity or religion in the FGDs due to logistical difficulties of recruiting and hosting a FGD with multiple layers of stratification, interviewers sampled women across a mix of different ethnicities and religions. Healthcare providers were sampled from the study facilities based on their cadre, and across a mix of older/more experienced and younger/less experienced. Facility administrators were sampled from the study facilities.

### Study instruments

All instruments were semi-structured discussion guides, fostering comparability across IDIs/FGDs and allowing participants to guide the discussion based on their experiences. In the FGDs, women were not asked to disclose their individual experiences of mistreatment, but were asked to speak about “women like them” or an anonymous friend/family member who has experienced this treatment. Likewise, healthcare providers and administrators were not asked to disclose instances where they mistreated a woman; rather, they were asked to speak about mistreatment they witnessed during their work. The following domains of interest were explored with all participants, with small differences to adjust specific questions based on type of participant: (1) expectations of care during childbirth at health facilities; (2) experiences and perceptions of mistreatment during childbirth; (3) decision-making processes to deliver at a facility; (4) views of acceptability of mistreatment during childbirth; (5) perceived factors influencing mistreatment of women during childbirth; and (6) treatment of staff by colleagues and supervisors.

### Data collection and management

Research assistants were female Masters of Public Health graduates with training in qualitative research and maternal health. All research assistants were from Ibadan, Nigeria and underwent a 2-day training and piloting workshop in Abuja prior to commencing data collection. Eligible individuals completed a written consent form prior to participation. All FGDs and IDIs took place in a private setting with only participants present, were audio recorded, lasted 60–90 min and were conducted by research assistants. Participants received 2000 Naira (approximately $10 USD) to compensate for their transportation cost and a refreshment. Data were collected from March to June 2015, until thematic saturation was reached. Transcription, translation and recording of field notes occurred in parallel, and transcripts were shared and reviewed on an on-going basis to ensure data quality. IDIs and FGDs conducted in English were transcribed in English, and those conducted in a local language (Pidgin English, Hausa, Igbo or Yoruba) were translated and transcribed simultaneously by the research assistants. De-identified transcripts were stored on a password protected computer.

### Data analysis

This analysis employs a thematic analysis approach, as described by Braun and Clarke [45]. Thematic analysis is inherently a flexible method, and is useful for identifying key themes, richly describing large bodies of qualitative data and highlighting similarities and differences in experiences [45].

After transcription, line-by-line coding was performed on a subsample of transcripts by two independent researchers to develop an initial thematic framework. These codes emerged naturally from the data and were initially structured as “free codes” with no established link between them. Free codes were synthesized with questions from the discussion guide and systematic review findings [29] into a coding scheme transferable to other transcripts. The coding synthesis yielded a hierarchical codebook to explore higher-level concepts and themes and organize the codes into meaningful code families. Reliability testing of the codebook was conducted in two stages: (1) two researchers jointly coded three transcripts, one from each type of participant; and (2) two researchers independently coded two transcripts and discussed coding decisions until consensus. After reliability testing, the final codebook was developed, which includes the structure of code families, code names, definitions, and an example of proper use. All transcripts were subsequently coded using Atlas.ti [46]. Memos were used to collate emerging thoughts, highlight areas of importance and develop ideas throughout the analysis process. A subset of the coded transcripts was reviewed by an independent researcher to check reliability of the coding.

Transcripts were organized according to meaningful “primary document families” in Atlas.ti [46], a method of organizing groups of transcripts based on common attributes, and used to restrict code-based searches or to filter coding outputs [47]. Primary document families consisted of: (1) type of participant; (2) facility/catchment area; and (3) religion. Output and reports were generated for specific codes using Atlas.ti [46] and filtered by primary document family where appropriate. Data from these reports and output were further synthesized into meaningful sub-themes, narrative text and illustrative quotations to draw connections between recurrent patterns and themes. These themes were interpreted within the context of the study and the typology of mistreatment during childbirth developed from the systematic review [29]. A 4-day data analysis workshop was also held with the research assistants, local investigators and WHO study team to interpret the findings in the Nigerian context.

Throughout this iterative analysis process, the research team considered questions of reflexivity, including identifying and reflecting on assumptions and preconceptions regarding what constitutes mistreatment, exploring emergent findings, and considering the research relationship.

### Technical and ethical approvals

Scientific and technical approval was obtained from the World Health Organization Human Reproduction Programme (HRP) Review Panel on Research Projects (RP2), and ethical approval was obtained from the World Health Organization Ethical Review Committee (protocol ID, A65880) and the Federal Capital Territory Health Research Ethics Committee in Nigeria (protocol ID, FHREC/2014/01/72/28-11-14).

This paper is reported according to the consolidated criteria for reporting qualitative research (COREQ) guidance [48].

## Results

A total of 84 IDIs and 4 FGDs are included in this analysis: 41 IDIs and 4 FGDs with women, 17 IDIs with nurses/midwives, 17 IDIs with doctors and 9 IDIs with administrators. Table 2 reports sociodemographic characteristics of participants: women of reproductive age, and Table 3 reports sociodemographic characteristics of participants: healthcare providers and administrators. Three eligible participants declined to participate: one administrator refused to give an audio-recorded interview, one woman did not have sufficient time to be interviewed, and one woman needed her husband’s permission but he was unavailable. We present an overview of preferences for childbirth in Abuja, followed by an overview of the context of mistreatment in this setting, and specific experiences of physical abuse, verbal abuse, stigma and discrimination and neglect, where data was richest, and solutions to reduce mistreatment as proposed by participants.

**Table 2:** Sociodemographic characteristics of participants: women of reproductive age

	IDIs (*n* = 41)	FGDs (*n* = 4 FGDs^a^)
Age (years)		
20–24	2	7
25–29	12	11
30–34	14	9
35–39	9	5
40+	4	2
Marital status		
Single	0	0
Married	40	33
Divorced/Widowed	1	1
Location		
Urban	6	0
Peri-urban	21	34
Rural	14	0
Religion		
Christian	21	26
Muslim	20	8
Ethnicity		
Yoruba	13	9
Igbo	6	6
Hausa	2	1
Idoma	1	1
Igala	4	7
Tiv	2	0
Urhobo	0	4
Other^b^/missing	13	6
Education		
None	1	4
Primary	1	1
Secondary	18	21
Tertiary	21	11
Employment		
Business/private sector	5	3
Civil servant	3	2
Hair dresser	2	5
Housewife	10	8
Tailor	5	0
Teacher	4	5
Trader	7	11
Other	5	0
Number of living children		
0–1	9	8
2–3	17	20
4–5	13	4
6+	2	2

^a^Three FGDs conducted with 8 women, one FGD conducted with 10 women

^b^“Other” includes Akwa-ibom, Angas, Ebira, Igede, Katarf, Ogori, Zuru, Akoko Edo, Bekwarra, Edo, Isoko, Ogoja

**Table 3:** Sociodemographic characteristics of participants: healthcare providers and administrators

	Nurse/midwives *n* = 17	Doctors *n* = 17	Administrators *n* = 9
Age (years)			
30–39	7	5	0
40–49	5	10	3
50+	5	2	6
Marital status			
Single	0	0	0
Married	15	17	8
Widowed	2	0	1
Gender			
Female	17	5	7
Male	0	12	2
Years of experience			
0–4	0	2	0
5–9	2	3	0
10–15	4	6	0
15+	11	6	9
Hospital			
Urban facility	8	9	5
Peri-urban facility	9	8	4

Traditionally, women in the North central region preferred home birth with a traditional birth attendant and family members present. This traditional model provided one-to-one care and support for the woman; however “anything can happen” if a woman gives birth at home [Woman IDI, 25 years old, rural], and if complications arise during a home birth, traditional birth attendants may not know how to cope. As women become more educated and “enlightened,” they tend to give birth at the hospital, but barriers to hospital attendance still exist, including financial cost, long distance and fear of mistreatment. Facility-based birth is perceived as normal in urban and peri-urban areas, where women believe that health facilities provide safe and effective care by trained staff and ensure safe childbirth and proper management of the mother and baby. Although the government subsidizes care provided at public hospitals, women commonly believe that they will be insulted and poorly treated if they attend there. Therefore, women with the means to pay for services may prefer to give birth at private hospitals, where they perceive they will be treated with respect since they are paying customers.

### Context of mistreatment in Abuja

While some participants described positive birth experiences where women were “well cared for” by the “helping hand” of the healthcare providers, both women and healthcare providers spontaneously brought up the topic of mistreatment, illustrating negative experiences that they had faced, witnessed or had heard about from others. Healthcare providers disclosed both scenarios where they felt that they had perpetrated mistreatment and where they witnessed a colleague mistreat a woman. Women and providers proffered explanations for why this situation occurred, and generally viewed them as by-products of an overstretched health system, rather than isolated events of intentional abuse. For example, a doctor from an urban facility explained that what women experience as neglect or abandonment by a healthcare provider may actually be a consequence of an understaffed facility:

*Respondent (R): If a midwife is already delivering a baby and another patient is calling for her attention you know she will not be able to attend* [to her] *at that particular time is that not so? No but to that patient she might feel that she has been treated wrongly isn’t it? But we know that that is far from that* [IDI male doctor, 42 years old, urban facility]

Healthcare providers described challenges faced on the labor ward, including “disobedient,” “uncooperative” and “unruly” women who made providing supportive care and “pampering” difficult. A doctor likened the labor ward to a war zone and explained that “*in the warfront you don’t pamper; when you are at war, you are at war*” [IDI male doctor, 40 years old, peri-urban facility]. Women sometimes lashed out at the healthcare providers, but explained that it was in retaliation for the poor treatment that they received:

*R: The labor started, they carried the woman to hospital, as they reached the hospital, they thing hooked the woman* [the labor pain hurt]*, so the woman was shouting and crying. That nurse, immediately, when she reach there, she gave the woman “baa!” (slap). Hey! The woman was just looking at her like ‘please you don’t know what is wrong with me and you slapped me, okay thank you.’ As the woman deliver, she said, she did everything for her. As the woman wanted to go…wanted to leave the hospital, the woman called the nurse, “please I want to see you”, she gave her (nurse) “fiam!” (slap). She said ‘the thing you did to me, that is what I did back to you.’* [FGD woman, 41 years old, urban]

Healthcare providers suggested that adolescents, primiparas and women of lower socioeconomic status may be more vulnerable to mistreatment, as healthcare providers may judge them for being pregnant too young, or they are unaware of what to expect during childbirth and appeared ill-prepared to engage with the health system. Furthermore, women who have not arranged to give birth in that facility (e.g.: unbooked for delivery) may be mistreated more often, as the lack of records contributes to a stressful environment for healthcare providers. These women were blamed for their lack of preparedness, even though providers were aware that they were more likely to be from disadvantaged backgrounds compared to women who had booked at that hospital for delivery.

A minority of healthcare providers, particularly doctors, believed that mistreatment does not occur in their setting. These doctors felt that women were dramatizing stories based on popular culture because they “watch all this film”, and were “exaggerating”, when in reality the healthcare providers “are professionals here, we don’t get angry, we only give professional advice.” These providers had the impression that because some women were unable to give detailed specifications of the mistreatment that they experienced, they were untruthful. However, women in this setting often do not have a forum for providing feedback on their experience or voicing experiences of mistreatment.

Understaffing and overcrowding on the labor ward can create a stressful work environment. Providers may “snap” or can be “wicked” in part due to the stresses in the work environment. These conditions contribute to healthcare providers’ feelings of impulsivity, lower tolerance for aberration, and exhaustion, and can contribute to transference of aggression to the woman. Working in these conditions may cause healthcare providers to “*not show the courtesy that is required of a health worker towards their client*” [IDI female administrator, 55 years old, urban facility]. A woman acknowledged that overworked and stressed healthcare providers were “*not computer, they are not engine, they get tired…it could lead to it* [mistreatment] *because when you are seeing the crowd alone…you are confused, you don’t even know where to start*” [IDI Woman, 34 years old, peri-urban].

However, several nurses believe that they usually have enough staff on a shift to cope with the needs of the hospital, and there is no excuse for how women are treated. Even when the facility is not overcrowded, it is in some healthcare providers’ nature to be rude:

*R: I tell you some of these times, nothing is happening, it’s not overcrowding, it’s not work… too much work, at times it happens! So many times it’s because of the bulk of the work but at times these things just happen even when the place is really calm*. [IDI female nurse, 39 years old, peri-urban facility].

### Physical abuse

Many healthcare providers and women provided detailed scenarios where women are slapped or beaten during childbirth, and commonly believed that slapping was used to ensure positive health outcomes for the woman and the baby. For example, if a woman closed her legs during childbirth, health workers would slap the woman to “encourage” her or give her the “strength” to “open up and deliver well”.

*R: If the woman is not cooperating. Like, you have your legs apart, the baby’s head is out, you understand, so and you are now trying to pull your legs back together. The, the nurse that could be taking delivery at that time could be so agitated. Thereby, just palm the woman on the thing* [slap the woman on the thigh] *that “open up” so that she can actually deliver the baby. It is not really mistreatment. It is helping the woman indirectly.* [IDI female doctor, 36 years old, urban facility]

Slapping is used to gain compliance and cooperation from a woman, and was often not considered to be mistreatment by women or healthcare providers provided that it was not done out of “malice”. Although women reported that it hurt, some women believe that healthcare providers would not act outside of the best interests of the woman, and would blame the other women for “trying to kill their babies”.

*I: Okay, how you feel when they slap you?*

*R: The slap, I felt bad but I, when I deliver the baby, I know that they help me, I didn’t carry it in mind and go again, because if that baby die I lose, if I myself die, we all lose, so at least I prefer that slap than I miss the baby* [FGD Woman, 35 years old, peri-urban]

*R: But just what I’m telling you, it’s just that if it is slapped during labor, it depends on what happened now. Just you know I’ve told you one instance, a woman is going, the head is out, she’s closing her leg. If you were in my shoe, what would you do? Will you leave her to kill the baby? No answer me. Will you leave her to kill the baby?* [IDI male doctor, 52 years old, peri-urban facility]

However, other women felt disempowered and experienced both physical and psychological pain that a healthcare provider would beat her: *“let them stop that shouting that they do, even beat they shouldn’t beat women, they should stop beating women for, during labor”* [FGD Woman, 29 years old, peri-urban]. These experiences took a toll on the woman both emotionally and physically: “*they slapped me, the five finger* [mark] *appear till when I reach home…I don’t even want to remember that past…she was supposed to pet me as far as it’s my first experience*” [FGD Woman, 30 years old, peri-urban]. Women felt that words of encouragement and a clear explanation of what the healthcare provider expected from the woman during childbirth would mitigate the need to slap her.

*R: Yes, I would, I pass through through it also. They beat me enough…Yes, they beat me hard, so hard that at the end of the whole thing…I find out that if I don’t push, I may end up dying or the baby may end up dying.*

*I: So how did you feel when they were beating you?*

*R: I was like telling them, “abeg now nurse now, take it easy, is not my fault, you see that is painful.” …I was just pleading with them, because I know them, they were also fed up with me. Do you understand me? I know they did their best. I know at that point in time, they were trying their best to save me. But because of* [the beating] *I do not have strength.* [IDI Woman, 31 years old, urban]

*R: Maybe a woman is pushing and she is not cooperative…we are not supposed to use our hand … to beat the woman. The way am taught…there are better ways to communicate to her… But sometimes, you see midwives beating patients, ‘you want to kill the baby!’ Pow! Pow!! Pow!!!* [IDI female nurse, 36 years old, urban facility]

Another method healthcare providers sometimes used to control a woman during childbirth was physically tying the woman to the delivery bed with ropes.

*R: I was the only midwife on duty, that time we used to work alone, just one person on duty, on night duty with the attendant, this lady, this lady came in, it’s her first time, she’s a primi, she was fully dilated! But no way! She will rather get up and stand! When she starts having contractions she will climb up the couch and remain there, so eventually when we were able to bring her down, I had to call her relations, you understand? Her relations and I had to call her relations and then bring the bed and put still ropes to hold her legs* [IDI female nurse, 39 years old, urban facility]

### Verbal abuse

Women described healthcare providers shouting, criticizing, insulting and speaking harshly during their childbirth. Rudeness was pervasive, and women felt that healthcare providers “*don’t care about human life*”, “*insult people like they’re not a human being*” and “*will maltreat you like a slave*”. A woman who gave birth in the peri-urban hospital explained that when she arrived for childbirth, the midwife said “*oya, go outside goat…Ehn see this goat, go outside, it’s not yet time, it’s not time, what are you doing here, you are disturbing me*” [IDI Woman, 29 years old, peri-urban]. Women were yelled at for not bringing all of the supplies needed to conduct the birth (e.g.: gauze, cotton, gloves, bed sheet) and for not complying with the healthcare providers’ demands.

*R: You know is very, very common with general hospitals, some can be very, very rude. The way they talk to you sometimes as if they are not being sensitive to your situation. You understand? You go and you want to seek for help, the way they…sometimes you see mothers blinking their faces and they will be crying. You understand? You don’t put their, themselves in your shoes. Talk to you anyhow, you know. Make you feel less important because you’ve come to general hospital…*

*I: Does it occur often or is something that is just rare?*

*R: Is something that is in their blood. Not rare* [IDI Woman, 31 years old, urban]

Healthcare providers also made judgmental comments about a woman’s sexual history, chastising them that they enjoyed having sex, but now the healthcare provider had to deal with the consequences of the resulting pregnancy and childbirth.

*R: Like when they insult you, “am I your husband? When your husband was doing it, it use to sweet you, but now you are disturbing us with your noise”. When you hear that kind of insult, even when they come to attend to you, you’ll be feeling shy, anything you want to do sef, when they say “okay, spread your leg”, you will be feeling shy to even spread your leg because they have already insult you that when your husband was doing it* [FGD Woman, 25 years old, peri-urban]

When they were verbally abused, women felt that they were more “vulnerable” and had reduced agency to communicate with the healthcare provider or to complain about poor services rendered. A woman who felt disrespected during her childbirth said “*I don’t have anything to say, is only God that will help me in this condition now because I don’t have the power*” [IDI Woman, 27 years old, rural]. Similarly, women’s cries of pain during labor were silenced by healthcare providers, who felt that women should be silent while they give birth. Healthcare providers confirmed that verbal abuse is common, and explained that they felt “agitated,” “irritated” and “annoyed” when women did not “cooperate” with their demands, but that they were not intentionally trying to harm the woman. After an outburst, they would often feel badly about raising their voices, and apologize to the woman.

### Stigma and discrimination

Midwives and doctors explained that HIV-positive women may fear discrimination and hide their status from healthcare providers to prevent such discrimination from occurring. This may put health workers and their babies at risk of contracting HIV if appropriate protection is not used. Women also felt discriminated against when they were of different religions, ethnicities or from low socioeconomic status.

*R: The health workers try but they can do better…They’re not very nice, to women, okay especially if they look at the woman and they have some bias, yeah, they are like where is she from, you know that kind of thing, or she doesn’t look so clean, she looks dirty or something, most people don’t look at their patients with the human face, you look at people and you are already judging them, I think that if a woman comes, for example, she’s coming from a squalid background, she’s dirty, you can actually give her a bath and make her feel nice and then you bring her back in and continue what you’re doing* [IDI female doctor, 36 years old, urban facility].

R: *If you have an educated person…they reason better than these non-educated people. So if you have an educated person, you don’t have to clash with them because they reason very well. These uneducated people, they’re poor, stupid, ignorant, nothing. They anger you.You’re trying to save his wife; he doesn’t understand what you’re talking about.* [IDI male doctor, 42 years old, peri-urban facility]

### Neglect and abandonment

Women commonly felt neglected during labor and felt unable to summon healthcare providers when needed. They were seldom monitored during labor, and if complications arose, such as excessive bleeding, it was difficult to get the attention of a healthcare provider. Providers confirmed that in some cases, they felt overworked and did not take the appropriate time to address the needs of the woman:

*R: Like some patients they are not good…just rush in and want you to leave what you are doing or… come and attend to them. You feel so irritated and you talk to them any how or you will even send them away that you are not going to attend to them… I have done it before too. Like 4 years ago we used to have more than 13 deliveries in a night and it will just be 2 nurses on duty, by the time you are handling over in the morning you will see that your legs are shaking, and they will now bring one unbooked patient for you to leave what you are doing to come and attend, your head will be banging and you won’t even know when you will tell them to go to hell anywhere they want to go let them go.* [IDI female nurse, 40 years old, peri-urban facility].

Furthermore, public facilities are often overcrowded, with not enough beds for women. As a result, women are sometimes forced to give birth on the floor, and without the support of a healthcare provider. One woman described another woman giving birth on the floor when she arrived at the hospital due to insufficient bed space:

*R8: [the hospital has] four beds, but the population there people that want to deliver they are up to 8.... so as I was standing there, one Gbagyi woman they just hold the woman, the woman was even holding the baby, before they will check the woman, the woman just lie on the floor and deliver.* [FGD Woman, 31 years old, peri-urban].

### Violations of privacy

The structure of the facility contributed to mistreatment, as some women felt that their privacy was violated by the poor design of the labor wards, where women would be exposed to other patients, their families and providers. Delivery rooms contained several beds with no partitions between them, and if curtains were available, they were tattered or not closed properly. Windows were broken and lacked curtains to shield women from passersby.

*R: Even when I was delivering many people are passing by, they were looking at me. It’s supposed to be enclosed but they did not repair everything that they suppose, like window, everything have spoilt and they did not do it, they did not repair…I mean according to our religion is not allowed, everybody were seeing our naked. How the baby will come out, so…I was annoyed.* [IDI Woman, 31 years old, peri-urban].

### Impact of mistreatment on care-seeking

Experiencing mistreatment could be “destabilizing” for women who are often vulnerable during childbirth:

*R: The attitude of the health workers can influence on a woman either negatively or positively…if they teach you well, encourage you, it will give you that confidence, you understand, but if they are rude and harsh, it will destabilize you and add to your problem.* [IDI Woman, 29 years old, urban]

Women feared mistreatment during facility-based childbirth to the extent that they sometimes avoided attending the facility altogether: “*women they are dying at home because of they are fearing to go to hospital because of the way nurse and doctor they are treating them*” [FGD Woman, 30 years old, peri-urban]. These women believed that they would be better supported during a home birth, and that they will be mistreated if they attend the hospital.

### Solutions to improve the treatment of women during childbirth

At the end of the IDIs and FGDs, healthcare providers and women were asked what could be done so that women were treated better during labor and childbirth. Both groups noted that solutions to improve how women are treated during childbirth will need to be multifaceted and multidimensional across different levels of the health system, from provider sensitization and training through physical infrastructure strengthening. Training should be provided on how to give respectful and compassionate care, to reorient providers suffering from “compassion fatigue” and “put yourself in the woman’s shoe”. This training should be integrated with coping mechanisms for working in stressful environments, increase provider motivation and techniques for improving patience, tolerance and endurance. The physical structure of the facilities should be adapted to ensure that they are properly equipped to handle deliveries, such as providing adequate private space for women to give birth, designing the space that is compatible to labor companions, and providing clean toilet and washing facilities for women. Both women and healthcare provider suggested improving salaries of providers working in public facilities and increasing staffing to alleviate stress and pressure on the providers. There should also be facility-level redress mechanisms for women to express dissatisfaction or satisfaction with the services rendered. Creating a forum to promote engagement between healthcare providers and women to manage expectations could ultimately reduce provider stress as it would allow healthcare providers to better explain to women and their families what supplies to bring with them to the hospital, and women to understand why such supplies are needed, and in what circumstances a woman may need to pay for services she receives.

## Discussion

This study explored women and healthcare providers’ experiences and perceptions of mistreatment during childbirth in the North central region of Nigeria, and provides the first known qualitative evidence of mistreatment during childbirth in Nigeria. The findings suggest that across urban and peri-urban/rural settings, age groups and religions, women experience and providers acknowledge mistreatment during childbirth. Women and providers reported experiencing or witnessing physical abuse such as slapping, being tied to a delivery bed, and detainment in the hospital and verbal abuse, such as shouting at, intimidating, and threatening women with physical abuse. In some cases, women overcame tremendous barriers to reach a hospital, only to give birth on the floor, unattended by a healthcare provider. Participants in this study identified three main factors contributing to mistreatment: poor provider attitudes, women’s behavior, and health systems constraints. Slapping a woman during childbirth was viewed as a means by which to ensure a positive outcome, and that women provoked healthcare providers when their disobedience endangered her baby. Systemic physical resource and staffing constraints contribute to a disabling work environment and propagate provider stress, and when providers cannot cope with this stress, they may transfer their aggression onto the woman herself.

In this study, both women and providers blamed mistreatment during childbirth on a disempowering health system where providers are overworked and facilities are understaffed and overcrowded. This explanation parallels other literature in the field [27, 28, 49]; however mistreatment cannot be blamed solely on the health system. Another publication resulting from this study explored social norms and acceptability of mistreatment during childbirth in Nigeria, and found that both women and healthcare providers considered physical and verbal abuse as acceptable and appropriate measures to gain compliance from the woman and ensure a good outcome for the baby [50]. Furthermore, in Nigeria, women and their families are unable to express their satisfaction or dissatisfaction with services rendered, thus hindering the ability to engage with users and improve the quality of care. When there are no ramifications for poor quality of care and women’s concerns are suppressed or ignored, there is little incentive to foster change. However, consistent and targeted audit and feedback, including feedback from women on their care experiences, can have a significant effect on improving healthcare providers’ compliance with a desired practice [51].

In many low- and middle-income countries (LMICs), including the two facilities in this study, women are denied labor companionship. However, a review by Hodnett and colleagues concluded that women who received continuous one-to-one support (either by a skilled healthcare provider, doula/birth educator, member of the woman’s social network, or a stranger with no special training in labor support) were less likely to have dissatisfaction and negatives views about the birth experience, intrapartum analgesia, instrumental vaginal birth, regional analgesia or a baby with a low five-minute Apgar score, and more likely to have spontaneous vaginal birth [52]. It is possible that the benefits of labor companionship could also extend to reduce experiences of mistreatment, as a labor companion could act as an advocate for the woman. However, key knowledge gaps exist, particularly regarding how to implement labor companionship in LMICs. A qualitative evidence synthesis would be useful to identify barriers and facilitators to the successful implementation of labor companionship and to better understand how and why companionship leads to improved outcomes [53].

A systematic review [29] and this study found that overcrowded and understaffed maternity wards fostered a high-stress work environment. Promotion of interventions to promote mindfulness and other stress-coping mechanisms may be useful management tools [54]. Finally, “pay for performance” approaches may be useful to motivate healthcare providers to deliver higher quality services [55].

This study was conducted in two facilities and facility-catchment areas in the Abuja metropolitan area, and may not reflect the experiences of women and healthcare providers across Nigeria. However, most health workers working in this area are trained in different regions of Nigeria, and their attitudes and practices are therefore shaped by their pre-service training. Interviews were conducted with women who had given birth any time during the previous year and may therefore have recall bias. Similarly, mistreatment is a difficult topic to discuss with providers, and providers therefore may have underreported such experiences, particularly for interviews with providers conducted in health facilities (social desirability bias).

## Conclusions

Moving forward, there are several critical next steps. First, findings from this study should be communicated to key stakeholders including providers and administrators at the study facilities. Such efforts should also demonstrate how physical resource and staffing constraints in the healthy system can have profound impacts on a woman’s birth experience. Women must be given a platform to voice their experiences of care, and tough discussions must be had with providers and policy-makers to unpack the uncomfortable topic of deliberate abuse versus unintentional neglect. Second, measurement tools to assess how often mistreatment occurs and in what manner must be developed for monitoring and evaluation. Third, global health leaders, researchers, advocacy groups and other key stakeholders must collaborate to develop a global definition of the mistreatment of women during childbirth. Such efforts are necessary to put the mistreatment of women during childbirth on the global agenda, especially in the context of Sustainable Development Goals 3 (ensure healthy lives and promote well-being for all at all ages) and 5 (achieve gender equality and empower all women and girls) [56]. Finally, any intervention to prevent mistreatment will need to be multifaceted, and researchers, implementers and policy-makers should consider lessons learned from related interventions, including audit and feedback [51], labor companionship [52] and stress-coping mechanisms for providers [54].

## Abbreviations

COREQ: Consolidated criteria for reporting qualitative research
DHS: Demographic and Health Survey
FGDs: Focus group discussions
FHREC: Federal Capital Territory Health Research Ethics Committee in Nigeria
HRP: World Health Organization Human Reproduction Programme
IDIs: In-depth interviews
LMICs: Low- and middle-income countries
RP2: Review Panel on Research Projects
TFR: Total fertility rate
